# Epidemiology and outcomes of invasive fungal infections in patients with mature T-cell and NK-cell lymphomas

**DOI:** 10.1080/07853890.2026.2720365

**Published:** 2026-08-26

**Authors:** Ming-Tao Tsai, Pao-Yu Chen, Tai-Chung Huang, Jann-Tay Wang, Wen-Chien Chou, Yee-Chun Chen

**Affiliations:** a Department of Medicine, National Taiwan University Cancer Center, Taipei, Taiwan; b Division of Infectious Diseases, Department of Internal Medicine, National Taiwan University Hospital, Taipei, Taiwan; c Division of Hematology, Department of Internal Medicine, National Taiwan University Hospital, Taipei, Taiwan; d National Institute of Infectious Diseases and Vaccinology, National Health Research Institutes, Miaoli, Taiwan; e School of Medicine, National Taiwan University College of Medicine, Taipei, Taiwan

**Keywords:** Immunocompromised hosts, invasive yeast infection, invasive mould infection, mortality, *Pneumocystis jirovecii* pneumonia

## Abstract

**Background:**

Invasive fungal infection (IFI) causes poor outcomes in haematological malignancies, but data in mature T-cell and NK-cell lymphomas (T/NKCL) are scarce. We aimed to define epidemiology and outcomes of IFI in this population.

**Materials and methods:**

This retrospective study enrolled adult patients with T/NKCL between 2019 and 2024. Proven and probable IFI were defined according to the 2020 EORTC/MSGERC criteria. Multivariable logistic regression was used to identify factors associated with IFI and death.

**Results:**

Among 203 patients, 43 (21%) developed 52 IFI episodes, including invasive yeast infections (IYI, *n* = 22), invasive mould infections (IMI, *n* = 15), and Pneumocystis jirovecii pneumonia (PCP, *n* = 15); eight patients experienced multiple episodes. Median time to IFI onset was 128 days for IYI, 455 days for IMI, and 62 days for PCP. In multivariable analysis, IFI was independently associated with male gender (aOR 3.22; 95% CI 1.32–8.72), high lymphoma Ann Arbor stage (aOR 3.58; 95% CI 1.46–9.75), gastrointestinal bleeding (aOR 11.32; 95% CI 2.61–57.13), and haematopoietic stem cell transplantation (aOR 5.96; 95% CI 2.51–14.67), while achievement of disease remission was associated with a reduced risk (aOR 0.36; 95% CI 0.15–0.81). IFI was an independent predictor of all-cause mortality (aOR 6.33, 95% CI 2.46–17.83) in multivariable analysis. In the adjusted time-dependent Cox model, IFI remained independently associated with lower survival (aHR, 9.53; 95% CI, 5.79–15.68).

**Conclusions:**

IFI is common and confers substantial mortality in patients with T/NKCL, with distinct pathogen-specific timing. These findings support early risk stratification and targeted preventive strategies in this high-risk population.

## Key Messages

- Invasive fungal infections occurred in one in five patients with mature T/NKCL.
- IFI predictors included male, lymphoma status, and treatment-related complications.
- IFI was associated with markedly increased mortality.

## Introduction

Invasive fungal infections (IFIs) remain a major cause of morbidity and mortality among patients with haematological malignancies [1], particularly those with acute leukaemia [2] and haematopoietic stem cell transplant (HSCT) recipients [3]. While the epidemiology and risk stratification of IFIs are well established in these high-risk groups, data on IFIs in lymphoid neoplasms—especially mature T-cell and natural killer (NK) cell lymphomas (T/NKCL)—remain limited.

According to the fifth edition of the World Health Organization classification of haematolymphoid tumours, T/NKCL comprises a heterogeneous group of predominantly aggressive lymphomas requiring intensive multimodal therapy, including cytotoxic chemotherapy, immunomodulatory agents, radiation therapy, and, in selected cases, autologous or allogeneic HSCT [4–6]. These treatments, together with disease-related immune dysregulation, expose patients to profound and often prolonged immunosuppression, suggesting a potentially distinct risk profile for IFI compared with other lymphoma subtypes.

Despite these biological and clinical considerations, IFIs in T/NKCL remain poorly characterised. Available evidence is limited to small retrospective series, including an Australian cohort in which fungal infections accounted for nearly one quarter of microbiologically documented infections, with several fatal cases, but only five IFI events were identified among 16 patients [7]. Consequently, the timing, drivers, and outcomes of IFI in T/NKCL are not well defined, and current IFI surveillance and prophylaxis recommendations may not adequately address the needs of this group. To address this knowledge gap, we conducted a 5-year retrospective cohort study to characterise the epidemiology and clinical spectrum of IFI, to identify factors associated with IFI development, and to evaluate its impact on mortality in patients with T/NKCL.

## Materials and methods

### Study design and population

This was a single-centre, retrospective observational study conducted at National Taiwan University Hospital (NTUH), a 2,500-bed tertiary medical centre in northern Taiwan. The study was approved by the NTUH Research Ethics Committee and conducted in accordance with the Declaration of Helsinki.

Adult patients (≥18 years) with a new diagnosis of mature T-cell or natural killer cell lymphoma (T/NKCL) between January 2019 and December 2024 were eligible. Patients who were not treated at NTUH or who had less than 6 months of follow-up were excluded, unless invasive fungal infection (IFI) or death occurred within this period. Diagnoses were histologically confirmed and classified according to the fifth edition of the World Health Organization classification of haematolymphoid tumours [4]. Eligible entities included mature T-cell and NK-cell leukaemias, primary cutaneous T-cell lymphomas, intestinal T-cell and NK-cell lymphoid proliferations and lymphomas, hepatosplenic T-cell lymphoma, anaplastic large cell lymphoma, nodal T-follicular helper cell lymphoma, peripheral T-cell lymphoma not otherwise specified, and Epstein–Barr virus–positive NK/T-cell lymphomas.

### Data collection

Baseline variables collected at lymphoma diagnosis included age, gender, comorbidity burden assessed using the Charlson Comorbidity Index (CCI) [8], lymphoma Ann Arbor stage and International Prognostic Index [9,10]. Disease- and treatment-related variables were collected longitudinally and included lymphoma-directed treatment regimens, use of corticosteroids, antibody-drug conjugate therapy (brentuximab vedotin) and haematopoietic stem cell transplantation (HSCT). Microbiological, radiological, and histopathological data related to IFI episodes were reviewed. For the risk-factor analysis of IFI development, baseline characteristics were defined at lymphoma diagnosis. Clinical events occurring during the disease course, including haemophagocytic lymphohistiocytosis, abdominal surgery, gastrointestinal bleeding, and neutropenia, were analysed as pre-IFI clinical events. In patients who developed IFI, these variables were considered present only if they occurred before the onset of the first IFI episode. In patients without IFI, these variables were assessed from lymphoma diagnosis to the last follow-up. Neutropenia was defined as an absolute neutrophil count <500/μL and was analysed as a clinical event during follow-up rather than as a baseline-only characteristic, and for patients with IFI, neutropenia was counted only if it occurred within 90 days before IFI onset. Granulocyte colony-stimulating factor was administered according to institutional practice for absolute neutrophil counts <0.5 × 10³/µL.

Frontline lymphoma-directed therapy was defined as the initial lymphoma-directed treatment administered after diagnosis with the intent to induce remission before response assessment, consolidation, maintenance, HSCT, or salvage therapy. This term was used throughout the manuscript to encompass systemic chemotherapy, concurrent chemoradiotherapy, and other lymphoma-directed frontline approaches.

### Antifungal prophylaxis

Antifungal prophylaxis was prescribed following multidisciplinary assessment by infectious diseases specialists and primary care haematologists, in accordance with national guidelines [11]. During allogeneic HSCT, micafungin (50 mg/day) was administered until neutrophil engraftment. Trimethoprim–sulfamethoxazole prophylaxis was generally provided during intensive chemotherapy and routinely after allogeneic HSCT. Posaconazole prophylaxis was routinely used in patients with acute graft-versus-host disease requiring systemic corticosteroids.

### Diagnostics and definitions of invasive fungal infections

Diagnostic evaluation for IFI was performed in patients with compatible clinical syndromes; routine surveillance in asymptomatic patients was not undertaken. The exception was serum *Aspergillus* galactomannan screening (GMAg), which was performed twice weekly after allogeneic HSCT for up to one year or until cessation of immunosuppressive therapy, per institutional practice. Additional diagnostic modalities included fungal cultures, microscopy, histopathology, cryptococcal antigen testing, polymerase chain reaction (PCR) assays for *Pneumocystis jirovecii*, and computed tomography of the chest and/or paranasal sinuses. Infectious diseases consultation was routinely obtained for suspected or confirmed IFI.

Only proven and probable IFI, defined according to the 2020 revised European Organization for Research and Treatment of Cancer/Mycoses Study Group Education and Research Consortium (EORTC/MSGERC) criteria [12], were included. IFI were categorised as invasive yeast infections (IYI), invasive mould infections (IMI), or *Pneumocystis jirovecii* pneumonia (PCP). GMAg was tested using the Platelia^™^ Aspergillus Ag assay (Bio-Rad, Marnes-la-Coquette, France). GMAg positivity was defined according to the 2020 revised EORTC/MSGERC consensus definitions as a single serum or plasma galactomannan index ≥1.0, or a bronchoalveolar lavage fluid galactomannan index ≥1.0. The date of IFI onset was defined as the first date on which diagnostic criteria were fulfilled. Catheter-related bloodstream infection (BSI) was defined by a differential time to positivity >2 h between peripheral and central line cultures.

### Outcomes

The primary outcome was all-cause mortality. Patients were followed from lymphoma diagnosis until death or study censoring on 31 December 2024.

### Statistical analysis

Continuous variables were summarised as medians with interquartile ranges (IQRs), and categorical variables were summarised as counts and percentages. Group comparisons were performed using Student’s *t* test or the Mann–Whitney *U* test for continuous variables and the χ^2^ test or Fisher’s exact test for categorical variables, as appropriate. Variables with *p* ≤ 0.20 in univariable analyses were entered into multivariable logistic regression models to identify factors associated with IFI development and all-cause mortality. Stepwise bidirectional selection was used to determine the final main model.

Two sensitivity analyses were performed to further evaluate the heterogeneity of IFI timing and to assess the potential influence of HSCT on IFI risk, respectively. Model A was restricted to early IFI, defined as IFI occurring within 1 year after lymphoma diagnosis. Model B focused on pre-HSCT IFI, with only IFI episodes occurring before HSCT counted as events. We also performed exploratory subgroup analyses according to major IFI categories. All additional models were analysed in the same manner as the main model.

Because IFI occurred during follow-up, its association with overall survival was evaluated using a Cox proportional hazards model with IFI treated as a time-dependent covariate. The model was adjusted for age, sex, aggressive histology, and IPI score. A Simon–Makuch plot was generated to visualise survival according to time-dependent IFI status.

Analyses were conducted using R software (R Foundation for Statistical Computing, Vienna, Austria). A two-sided *p* value <0.05 was considered statistically significant.

### Ethical considerations

The study was approved by the Research Ethics Committee of NTUH (IRB No. 202412044RINE), with a waiver of informed consent due to the retrospective design.

## Results

### Incidence and distribution of invasive fungal infections

**Figure 1.**
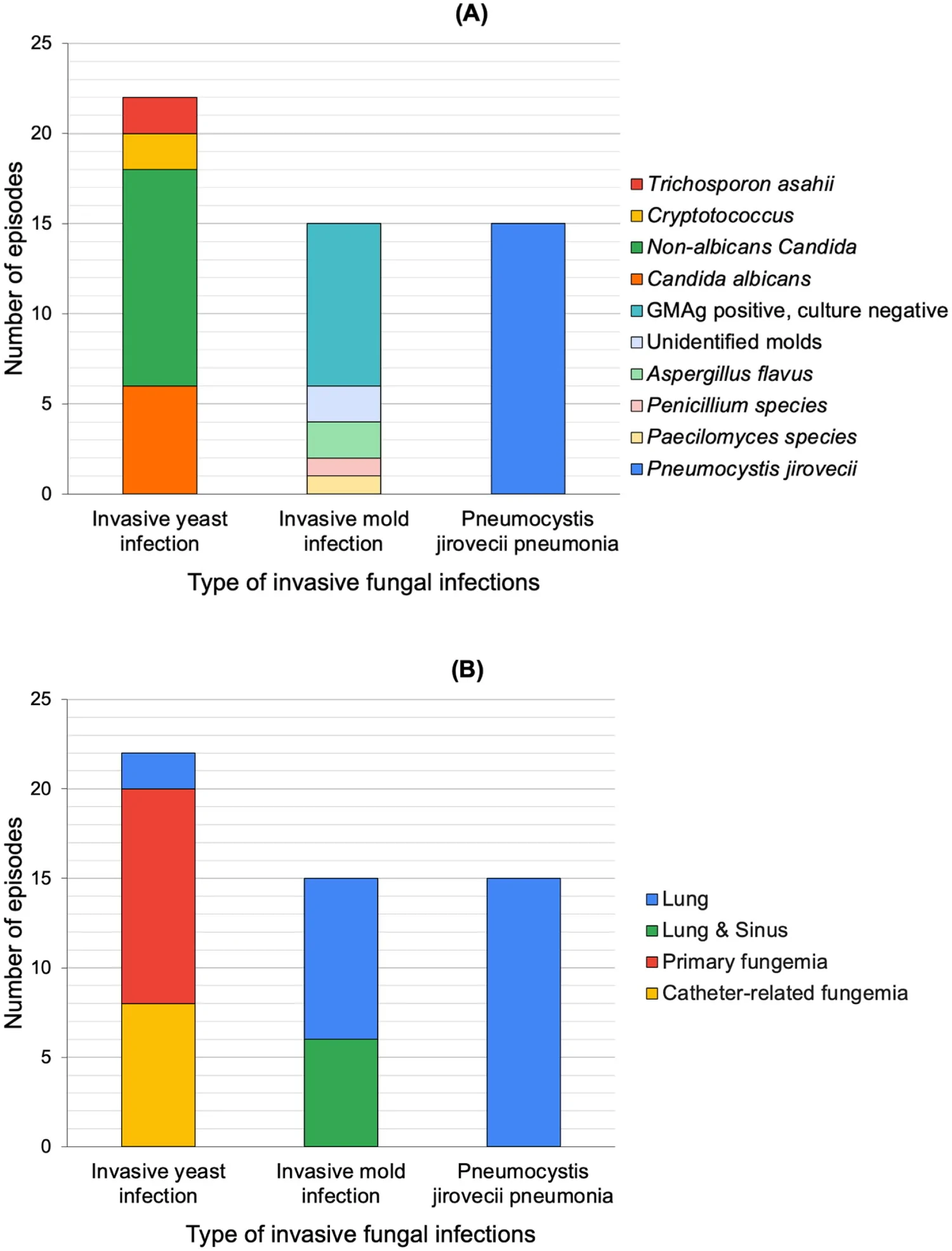
Distributions of etiologies (A) and clinical manifestations (B) of invasive fungal infections.

Among 203 patients newly diagnosed with mature T/NK-cell lymphoma, 43 (21%) developed 52 episodes of proven or probable invasive fungal infection (IFI), including 22 invasive yeast infections (IYI), 15 invasive mould infections (IMI), and 15 *Pneumocystis jirovecii* pneumonia (PCP) episodes (Figure 1A; Table 1). Eight patients (19%) experienced more than one IFI episode. Overall, 20 IFI episodes (39%) occurred during intensive care unit admission, including 8 IYI, 9 IMI, and 3 PCP episodes.

**Table 1.** Demographic and clinical characteristics among patients with and without invasive fungal infections.

Variable	All, *N* = 203	Without IFI, *N* = 160	With IFI^a^				
			Subtotal, *N* = 43	Invasive yeast infection, (22 episodes)	Invasive mould infection, (15 episodes)	*Pneumocystis jirovecii* pneumonia, (15 episodes)	*p* ^b^
Baseline demographic							
Age^c^	61 (43, 71)	62 (43, 72)	59 (48, 69)	63 (48, 70)	58 (42, 69)	58 (49, 67)	0.846
Male gender	121 (59.6)	88 (55)	33 (76.7)	15 (68.2)	12 (80)	14 (93.3)	0.010
Aggressive histology	155 (76.4)	113 (70.6)	42 (97.7)	21 (95.5)	15 (100)	15 (100)	<0.001
CCI ≥2	76 (37.4)	60 (37.5)	16 (37.2)	9 (40.9)	3 (20)	7 (46.7)	0.972
IPI ≥2	116 (57.1)	82 (51.3)	34 (79.1)	17 (77.3)	11 (73.3)	13 (86.7)	0.001
Stage ≥2	113 (55.7)	78 (48.8)	35 (81.4)	16 (72.7)	12 (80)	14 (93.3)	<0.001
Events during follow-up							
Haemophagocytic lymphohistiocytosis^d^	25 (12.3)	13 (8.1)	12 (27.9)	7 (31.8)	6 (40)	3 (20)	<0.001
Frontline lymphoma-directed therapy	141 (69.5)	100 (62.5)	41 (95.3)	20 (90.9)	15 (100)	15 (100)	<0.001
Disease remission after frontline lymphoma-directed therapy	108 (53.2)	95 (59.4)	13 (30.2)	6 (27.3)	3 (20)	5 (33.3)	<0.001
Corticosteroid^e^	140 (69)	101 (63.1)	39 (90.7)	20 (90.9)	14 (93.3)	14 (93.3)	<0.001
Brentuximab vedotin treatment	19 (9.4)	13 (8.1)	6 (14)	1 (4.5)	2 (13.3)	3 (20)	0.246
Abdominal surgery^d^	8 (3.9)	6 (3.8)	2 (4.7)	2 (9.1)	0 (0)	0 (0)	0.678
Gastrointestinal bleeding^d^**^,^**^f^	11 (5.4)	4 (2.5)	7 (16.3)	5 (22.7)	2 (13.3)	1 (6.7)	0.002
Neutropenia^d^**^,^**^g^	101 (49.8)	69 (43.1)	32 (74.4)	17 (77.3)	12 (80.0)	11 (73.3)	<0.001
Trimethoprim–sulfamethoxazole prophylaxis^h^	90 (44.3)	68 (42.5)	22 (51.2)	10 (45.5)	13 (87)	4 (26.7)	0.310
HSCT	33 (16.3)	19 (11.9)	14 (32.6)	4 (18.2)	8 (53.3)	5 (33.3)	0.001
Acute graft-versus-host disease^i^	8 (3.9)	2 (1.3)	6 (14)	2 (9.1)	5 (33.3)	0 (0)	0.001
Outcome							
Follow-up days	492 (195, 1182)	548 (225, 1368)	247 (81, 859)	169 (107, 542)	456 (146, 1090)	146 (59, 542)	0.005
All-cause mortality	83 (40.9)	49 (30.6)	34 (79.1)	19 (86.4)	12 (80)	12 (80)	<0.001

CCI, Charlson comorbidity index; HSCT, haematopoietic stem cell transplant; IFI, invasive fungal infection; IPI, International Prognostic Index.

^a^A total of 43 (21%) patients developed 52 episodes of proven or probable invasive fungal infections.

^b^Comparisons between patients with and without invasive fungal infections.

^c^Median (Q1, Q3); *n* (%).

^d^For patients with IFI, clinical events occurring during the disease course were analysed as pre-IFI clinical events, and were considered present only if they occurred before the onset of the first IFI episode.

^e^Corticosteroid dose ≥20 mg/day of prednisone or equivalent.

^f^GI bleeding requiring interventional radiology and/or esophagogastroduodenoscopy/colonoscopy.

^g^Lowest ANC <0.5 × 10^3^/µL.

^h^Four patients had previously received PCP prophylaxis for periods ranging from 23 to 336 days. PCP developed at a time span of 27, 113, 152, and 243 days after the last day on active prophylaxis.

^i^Acute graft-versus-host disease grade ≥3 classified according to the International Bone Marrow Transplant Registry Severity Index requiring systemic corticosteroid treatment.

Although most patients had aggressive histological subtypes (155/203, 76%), the burden of IFI was unevenly distributed across lymphoma entities (Supplementary Table 1). Specifically, primary cutaneous T-cell lymphomas accounted for one quarter of the cohort (51/203) but only 7% of IFI episodes. A similar discordance was observed in anaplastic large cell lymphoma. In contrast, EBV-positive NK/T-cell lymphoma, followed by nodal T-follicular helper cell lymphoma and peripheral T-cell lymphoma not otherwise specified, exhibited the highest proportions of IFI.

### Risk factors associated with invasive fungal infections

Compared with patients without IFI, those with IFI had higher proportions of male gender (77% vs. 55%), aggressive histology (98% vs. 71%), IPI ≥2 (79% vs. 51%), advanced stage of T/NKCL (≥II: 81% vs. 49%), haemophagocytic lymphohistiocytosis (HLH, 28% vs. 8%), frontline lymphoma-directed therapy (95% vs. 63%), corticosteroid exposure (91% vs. 63%), gastrointestinal bleeding (16% vs. 3%), neutropenia (74% vs. 43%), HSCT (37% vs. 12%), and aGVHD (14% vs. 1%) (*p* < 0.05 for all) (Table 1). They were less likely to achieve remission after frontline lymphoma-directed therapy (30% vs. 59%) (*p* < 0.001).

In multivariable analysis (main model), invasive fungal infections were independently associated with male gender (adjusted odds ratio [aOR] 3.22; 95% confidence interval [CI] 1.32–8.72), high lymphoma Ann Arbor stage (aOR 3.58; 95% CI 1.46–9.75), gastrointestinal bleeding (aOR 11.32; 95% CI 2.61–57.13), and haematopoietic stem cell transplantation (aOR 5.96; 95% CI 2.51–14.67), while achievement of disease remission was associated with a reduced risk (aOR 0.36; 95% CI 0.15–0.81) (Table 2). Corticosteroid exposure and brentuximab vedotin treatment were not significantly associated with IFI risk.

**Table 2.** Univariate and multivariable analyses for factors associated with invasive fungal infections.

	Univariable OR (95% CI)	*p*	Multivariable OR (95% CI)	*p* ^a^
Baseline demographic				
Age	1.00 (0.98–1.02)	>0.99		
Male gender	**2.70 (1.29–6.12)**	**0.008**	**3.22 (1.32–8.72)**	**0.014**
Aggressive histology	**17.5 (3.63–314)**	**<0.001**		
CCI ≥2	0.99 (0.48–1.97)	0.97		
IPI ≥2	**3.59 (1.68–8.41)**	**<0.001**		
Stage ≥2	**4.60 (2.10–11.2)**	**<0.001**	**3.58 (1.46–9.75)**	**0.008**
Events during follow-up				
Haemophagocytic lymphohistiocytosis^b^	**4.38 (1.81–10.6)**	**0.001**		
Frontline lymphoma-directed therapy	**12.3 (3.60–77.2)**	**<0.001**		
Disease remission after frontline lymphoma-directed therapy	**0.30 (0.14–0.60)**	**<0.001**	**0.36 (0.15–0.81)**	**0.017**
Corticosteroid	**5.70 (2.16–19.7)**	**<0.001**		
Brentuximab vedotin treatment	2.20 (0.78–5.78)	0.13		
Abdominal surgery^b^	1.25 (0.18–5.67)	0.79		
Gastrointestinal bleeding^b^	**7.58 (2.17–30.3)**	**0.002**	**11.32 (2.61–57.13)**	**0.002**
Neutropenia^b^	**3.84 (1.86–8.47)**	**<0.001**		
Trimethoprim–sulfamethoxazole prophylaxis	1.42 (0.72–2.80)	0.31		
HSCT	**3.58 (1.60–7.96)**	**0.002**	**5.96 (2.51–14.67)**	**<0.001**
Acute graft-versus-host disease	**12.8 (2.82–89.9)**	**<0.001**		

CCI, Charlson comorbidity index; HSCT, haematopoietic stem cell transplant; IFI, invasive fungal infection; IPI, International Prognostic Index.

^a^Main model: stepwise logistic regression; reference: those without invasive fungal infections.

^b^For patients with IFI, clinical events occurring during the disease course were analysed as pre-IFI clinical events, and were considered present only if they occurred before the onset of the first IFI episode.

For sensitivity analyses, we first restricted to those with early IFI, defined as IFI occurring within 1 year after lymphoma diagnosis. In this early-IFI analysis, the multivariable model demonstrated that male sex was associated with higher odds of early IFI (adjusted OR, 5.83; 95% CI, 1.77–27.47; *p* = 0.007), as were IPI ≥2 (adjusted OR, 4.75; 95% CI, 1.29–24.84; *p* = 0.033) and gastrointestinal bleeding (adjusted OR, 19.02; 95% CI, 3.63–134.28; *p* < 0.001) (Supplementary Table 2, Model A). Second, we performed another sensitivity analysis focusing on pre-HSCT IFI. By the multivariable model, IPI ≥2 (adjusted OR, 6.20; 95% CI 1.88–28.94; *p* = 0.007) and gastrointestinal bleeding (adjusted OR, 7.38; 95% CI 1.86–31.69; *p* = 0.005) were independently associated with pre-HSCT IFI (Supplementary Table 2, Model B).

A subgroup analysis with separate multivariable analyses for each major pathogen category was performed to look for pathogen-specific risk factors (Supplementary Table 3). Compared to those without IFI, those with IYI were associated with gastrointestinal bleeding and neutropenia. Those with IMI were associated with acute GVHD and gastrointestinal bleeding. Those with PCP were associated with male sex, haematopoietic stem cell transplant, and absence of trimethoprim–sulfamethoxazole prophylaxis.

### Disease manifestations, microbiological characteristics and timing of invasive fungal infections

The distributions of IFI etiologies and clinical manifestations are presented in Figure 1. Among IYI episodes, non-*albicans Candida* bloodstream infections predominated (12/22, 55%), including *Candida parapsilosis* complex (*n* = 3), *Candida glabrata* (*n* = 4), *Candida tropicalis* (*n* = 3), and *Candida krusei* (*n* = 2), compared with *Candida albicans* (*n* = 6) and *Trichosporon* (*n* = 2) bloodstream infections (Figure 1A). Central venous catheters were present in 19 episodes at fungemia onset, of which eight met criteria for catheter-related bloodstream infection (Figure 1B). Two additional patients were diagnosed with pulmonary cryptococcosis with antigenemia.

All 15 IMI episodes involved the lungs, and 60% had concomitant sinusitis. All patients were positive for serum GMAg; 2 had additional positive GMAg from bronchoalveolar lavage fluid. Identified pathogens included *Aspergillus flavus* (*n* = 2), *Penicillium* spp. (*n* = 1), *Paecilomyces* spp. (*n* = 1), and unidentified moulds (*n* = 2). Chest CT findings included consolidation (64%), nodules (50%), halo sign (29%), or cavitation (7%). About two-thirds showed bilateral involvement (64%), and half disclosed pleural effusion (50%). Mucosal thickening was present in three-fourths of sinus CT, performed in 8 patients, involving the maxillary (*n* = 4) or sphenoid (*n* = 2) sinus (Supplementary Table 4).

All 15 PCP episodes were confirmed by PCR (14 sputum and 1 bronchoalveolar lavage). Among 11 patients who received chest CT, 10 had bilateral ground-glass opacities, 5 had consolidations, and 1 had diffuse interlobular cysts. Notably, PCP occurred predominantly in patients not receiving active trimethoprim–sulfamethoxazole prophylaxis.

**Figure 2.**
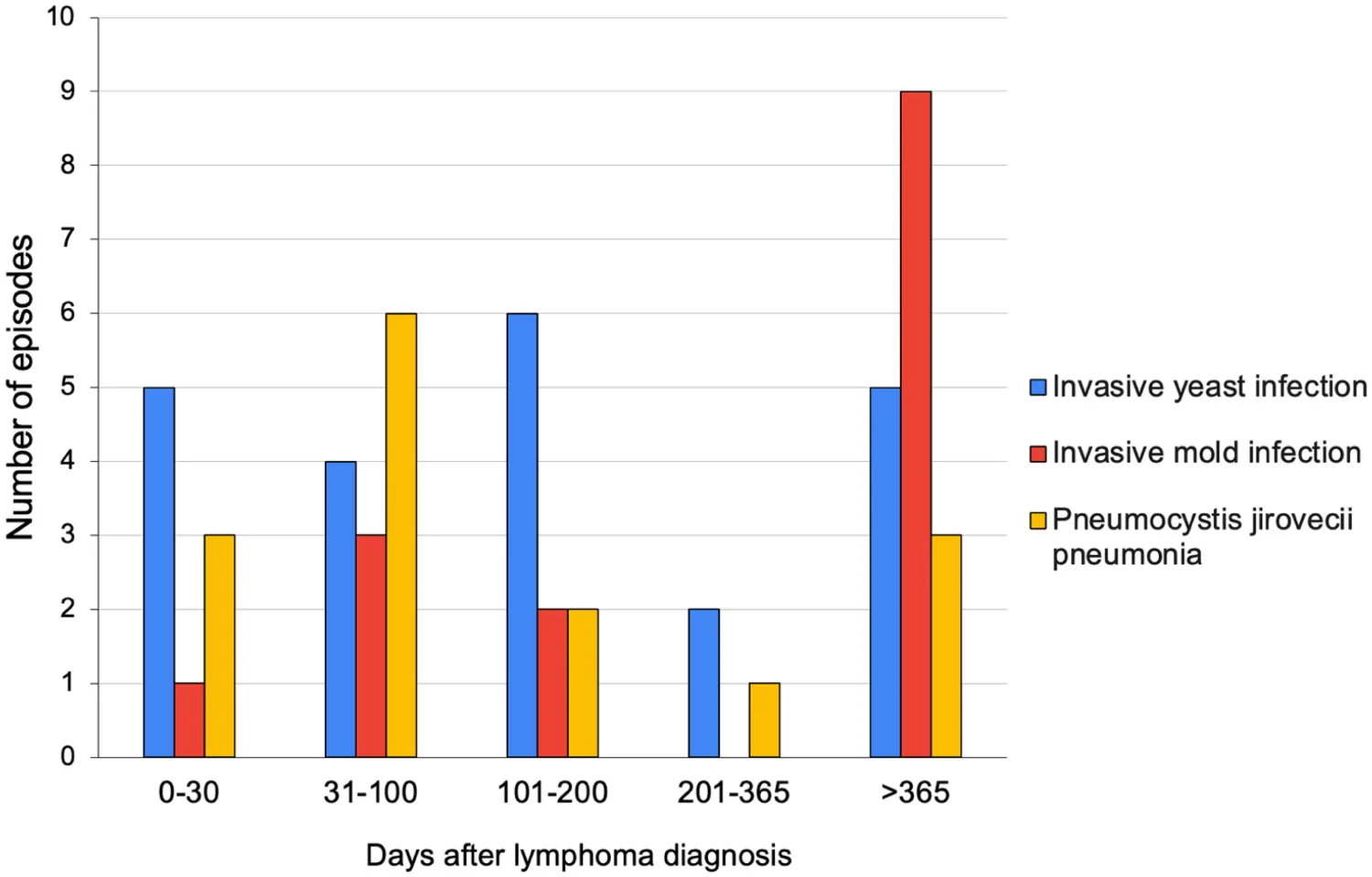
Time to onset of invasive fungal infection after lymphoma diagnosis.

The timing of IFI onset varied by pathogen group (Figure 2). Median time from lymphoma diagnosis to IFI onset was 62 days (IQR 37–345) for PCP, 128 days (IQR 49–342) for IYI, and 455 days (IQR 94–870) for IMI. PCP occurred early after lymphoma diagnosis, whereas IMI developed later in the disease course. IYI occurred throughout follow-up, with 40% of episodes arising during diagnosis or frontline lymphoma-directed therapy, within 3–36 days of the most recent treatment for lymphoma.

### Frontline lymphoma-directed therapy categories for mature T/NK-cell lymphoma

Frontline lymphoma-directed therapy categories for mature T/NK-cell lymphoma are summarised in Supplementary Table 5. Anthracycline-based CHOP-like regimens were the most common category, followed by asparaginase-containing regimens, concurrent chemoradiotherapy-based approaches, bendamustine-containing regimens, other regimens, and platinum-based regimens. In univariable analysis, frontline treatment category was associated with IFI development; however, regimen-specific estimates were imprecise because of small subgroup sizes and wide confidence intervals.

### Treatment outcomes and predictors of all-cause mortality

**Figure 3.**
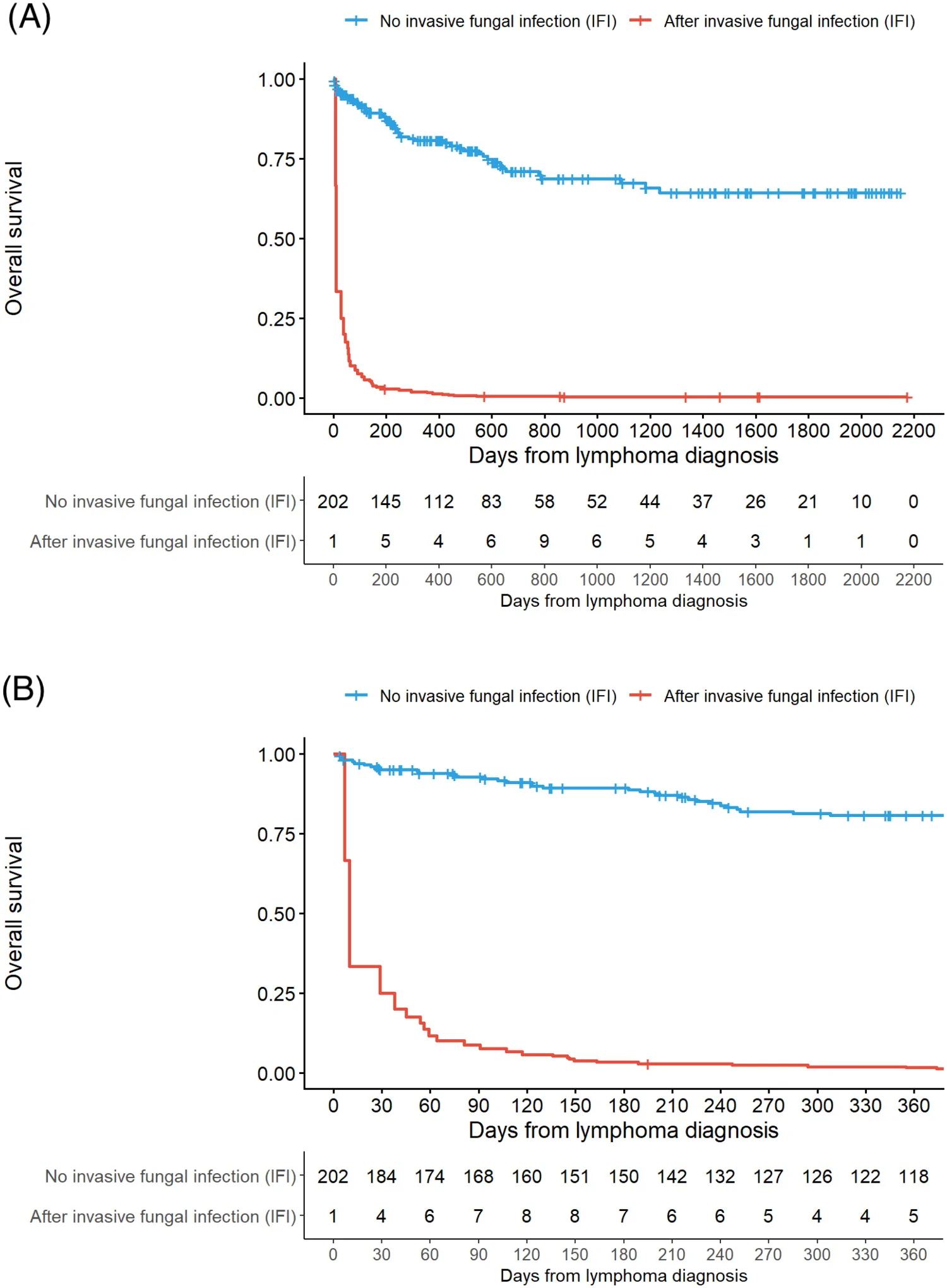
Simon–Makuch survival curve according to time-dependent invasive fungal infection (IFI) status within 5 years after lymphoma diagnosis (A) and within 1 year after lymphoma diagnosis (B). Patients contributed person-time to the ‘No IFI’ state before IFI onset and were reclassified to the ‘After IFI’ state at the time the first IFI episode occurred. The numbers at risk represent patients in each time-dependent state at each time point. One patient entered the ‘After IFI’ state on the date of lymphoma diagnosis because IFI diagnostic criteria were fulfilled on that date.

During follow-up, 83 patients (41%) died. Mortality was substantially higher among patients with IFI than those without IFI (79% vs. 31%; *p* < 0.001). In the adjusted time-dependent Cox model, IFI was independently associated with inferior overall survival (adjusted HR, 9.53; 95% CI, 5.79–15.68; *p* < 0.001). This association was visualised using a Simon–Makuch plot, in which patients were classified according to time-dependent IFI status during follow-up (Figure 3).

All eight patients who experienced multiple IFI episodes died. Short-term mortality following IFI was high. Fourteen-day and 42-day mortality were 50% and 68% for IYI, 27% and 60% for IMI, and 27% and 53% for PCP, respectively (Supplementary Table 6).

Regarding antifungal treatment, 73% of IYI patients received appropriate therapy. Among those with IMI, 93% received mould-active agents, and 27% required regimen modification due to intolerance or treatment failure. For PCP, 80% received targeted therapy. Detailed treatment regimens and outcomes are provided in Supplementary Table 6.

In multivariable analysis (Table 3), IFI independently predicted all-cause mortality (aOR 6.33; 95% CI 2.46–17.83). Other factors associated with mortality included older age (aOR 1.03 per year, 95% CI 1.00–1.06), aggressive histology (aOR 8.98, 95% CI 1.42–95.10), HLH (aOR 11.81, 95% CI 3.00–62.72), and IPI ≥2 (aOR 4.49, 95% CI 1.67–13.14). Notably, lymphoma achieving remission after frontline lymphoma-directed therapy was associated with significantly lower mortality risk (aOR 0.18; 95% CI 0.08–0.41).

**Table 3.** Univariable and multivariable logistic analyses for factors associated with all-cause mortality.

Variable	Overall *N* = 203	Alive, *N* = 120	Death, *N* = 83	Univariable OR (95% CI)	*p*	Multivariable OR (95% CI)	*p*
Baseline demographic							
Age^a^	61 (43, 71)	56 (40, 68)	64 (52, 75)	1.03 (1.01–1.05)	<0.001	**1.03 (1.00–1.06)**	**0.044**
Male gender	121 (59.6)	66 (55)	55 (66.3)	1.61 (0.90–2.89)	0.106		
Aggressive histology	155 (76.4)	74 (61.7)	81 (97.6)	25.2 (7.42–158)	<0.001	**8.98 (1.42–95.10)**	**0.036**
CCI ≥2	76 (37.4)	38 (31.7)	38 (45.8)	1.82 (1.02–3.26)	0.041		
IPI ≥2	116 (57.1)	45 (38.3)	71 (85.5)	9.86 (4.97–21.0)	<0.001	**4.49 (1.67–13.14)**	**0.004**
Stage ≥2	113 (55.7)	46 (38.3)	67 (80.7)	6.74 (3.56–13.3)	<0.001		
Events during follow-up							
Haemophagocytic lymphohistiocytosis^b^	25 (12.3)	3 (2.5)	22 (26.5)	14.1 (4.64–61.1)	<0.001	**11.81 (3.00–62.72)**	**0.001**
Frontline lymphoma-directed therapy	141 (69.5)	71 (59.2)	70 (84.3)	3.72 (1.90–7.69)	<0.001	0.32 (0.08–1.15)	0.09
Disease remission after frontline lymphoma-directed therapy	108 (53.2)	90 (75)	17 (21.7)	0.09 (0.05–0.18)	<0.001	**0.18 (0.08–0.41)**	**<0.001**
Corticosteroid	140 (69)	68 (56.7)	72 (86.7)	5.01 (2.49–10.8)	<0.001		
Brentuximab vedotin treatment	19 (9.4)	10 (8.3)	9 (10.8)	1.34 (0.51–3.47)	0.548		
Abdominal surgery^b^	8 (3.9)	4 (3.3)	4 (4.8)	1.47 (0.34–6.38)	0.596		
Gastrointestinal bleeding^b^	11 (5.4)	3 (2.5)	8 (9.6)	4.16 (1.16–19.4)	0.028		
Neutropenia^b^	105 (51.7)	48 (40)	57 (68.7)	3.29 (1.84–6.00)	<0.001		
Trimethoprim–sulfamethoxazole prophylaxis	90 (44.3)	47 (39.2)	43 (51.8)	1.67 (0.95–2.95)	0.075		
HSCT	33 (16.3)	15 (12.5)	18 (21.7)	1.94 (0.92–4.16)	0.084		
Acute graft-versus-host disease	8 (3.9)	2 (1.7)	6 (7.2)	4.60 (1.03–31.9)	0.046		
Any IFI	43 (21.2)	9 (7.5)	34 (41)	8.56 (3.96–20.2)	<0.001	**6.33 (2.46–17.83)**	**<0.001**

CCI, Charlson comorbidity index; HSCT, haematopoietic stem cell transplant; IFI, invasive fungal infection; IPI, International Prognostic Index.

^a^Median (Q1, Q3); *n* (%).

^b^For patients with IFI, clinical events occurring during the disease course were analysed as pre-IFI clinical events, and were considered present only if they occurred before the onset of the first IFI episode.

## Discussion

This study represents one of the largest cohorts of patients with mature T-cell and NK-cell lymphomas (T/NKCL) evaluated for invasive fungal infections (IFI) to date. Our findings demonstrated that IFI are frequent, affecting over 20% of patients, and are independently associated with a markedly increased risk of death. These results extend the current understanding of IFI beyond acute leukaemia and allogeneic haematopoietic stem cell transplantation (HSCT) and identify T/NKCL as an under-recognised population at substantial risk for IFI throughout the disease course. Historically, lymphomas have been considered an intermediate-risk group for IFI, lower than acute myeloid leukaemia or HSCT recipients [13]. However, our observed IFI incidence of 21% is considerably higher than that reported in most mixed lymphoma cohorts [14] and aligns with smaller series focused on T-cell lymphomas [7], underscoring that IFI risk in T/NKCL is likely underestimated in routine clinical practice.

Importantly, IFI risk was not uniform across T/NKCL histology subtypes. EBV-positive NK/T-cell lymphoma and nodal T-follicular helper cell lymphoma exhibited disproportionately high IFI burdens. Conversely, primary cutaneous T-cell lymphoma had a low incidence, consistent with its indolent clinical behaviour [15]. These findings suggest that IFI risk is heavily influenced by disease-specific characteristics, although subtype-based risk stratification requires further prospective validation.

Beyond histology, host and disease variables significantly influenced susceptibility. Notably, our finding of an association between IFI and male gender aligns with existing literature, which demonstrates a 1.5–3.5-fold increased risk among men–particularly for PCP compared to other IFIs [16]. This disparity may be partially driven by occupational and environmental exposure, as well as behavioral risks. Alternatively, sex-specific genetic and immunological factors have been postulated to drive this biological susceptibility [17]. The interplay between biological gender and IFI risk is highly complex, and warrants further mechanistic characterization. Additional factors associated with higher IFI risk by the main model (Table 2) included advanced Ann Arbor stage, HSCT, refractory disease and multiple treatment, which is consistent with prior studies [18]. Furthermore, both sensitivity analyses identified similar clinical factors—including patient characteristics, lymphoma stage, treatment response, and treatment-related complications—across different follow-up periods, supporting their relevance to IFI risk in patients with T/NKCL.

Regarding IFI subtypes, we identified gastrointestinal bleeding as a predictor for IYI, likely reflecting mucosal barrier injury, a well-recognised risk factor for invasive candidiasis [19]. Although trimethoprim–sulfamethoxazole prophylaxis has been shown to reduce PCP in non-HIV-infected patients [20], we did not observe a significant association between trimethoprim–sulfamethoxazole prophylaxis and IFI risk, likely reflecting the high prevalence of PCP prophylaxis in our cohort. Given the limited number of events within each IFI subgroup, these subgroup analyses should be considered as exploratory.

Furthermore, IFI risk in T/NKCL is driven by cumulative and dynamic immune dysfunction rather than a single dominant factor. Treatment-related factors likely compound disease-intrinsic immune dysfunction to further increase IFI susceptibility. Intensive multi-agent chemotherapy, repeated salvage regimens, and consolidative HSCT were commonly used in high-risk subtypes [6,21]. Consistent with previous studies [22], brentuximab vedotin was not independently associated with IFI risk, although event numbers were limited. Corticosteroid exposure, a recognised risk factor for invasive mould infections [23], was also not independently significant, likely due to its near-universal use and confounding with other immunosuppressive factors.

Outcomes following an IFI diagnosis were poor. Nearly 80% of patients with IFI died, and IFI remained strongly associated with all-cause mortality after adjustment for age, sex, aggressive T-cell lymphoma subtype, and IPI score. Importantly, this association persisted when IFI was modelled as a time-dependent Cox exposure, thereby reducing the potential for immortal-time bias inherent in conventional survival analyses based on ever/never IFI status. In contrast, achieving remission after frontline lymphoma-directed therapy was strongly protective, underscoring that effective control of the underlying malignancy is central to improving outcomes [18]. We further created a Sankey diagram (Supplementary Figure 2) illustrating dynamic risk for IFI throughout the T/NKCL disease course [24]. Patients who failed frontline lymphoma-directed therapy and proceeded to HSCT represented a particularly vulnerable group, experiencing a high cumulative burden of IFI and dismal survival, similar to prior reports linking disease refractoriness and multiple treatment lines to higher risks for IFI [18]. These findings highlight the urgent need for intensified surveillance and early diagnostic strategies in this setting.

This study has several strengths, including the large cohort of patients with T/NKCL evaluated for IFI, detailed characterisation of timing and risk factors, and the use of an internal comparator group. However, several limitations should be acknowledged. First, the retrospective design and heterogeneity of lymphoma treatment regimens introduced inevitable confounding. Second, IFI incidence may have been underestimated because fungal diagnostic testing, including serum β-D-glucan and other mycological assays, was not performed systematically throughout the study period. Third, the relatively small number of IFI events limited statistical power for subgroup analyses. Fourth, several clinically relevant variables, including haemophagocytic lymphohistiocytosis, abdominal surgery, gastrointestinal bleeding, and neutropenia, occurred during the lymphoma treatment course rather than exclusively at baseline. These variables were summarised as binary exposures rather than modelled as formal time-dependent covariates or analysed using landmark methods. Therefore, associations between these clinical events and IFI should be interpreted cautiously and hypothesis-generating. In addition, high early mortality limited the feasibility of landmark analysis. Finally, as this was an Asian cohort with a higher prevalence of EBV-associated and extranodal T/NKCL, generalisability to Western populations may be limited [25].

## Conclusions

Invasive fungal infections are frequent and highly lethal in patients with mature T-cell and NK-cell lymphomas. Distinct timing patterns were observed across invasive yeast, mould, and Pneumocystis infections. These findings highlight actionable opportunities for prevention and early recognition, particularly through consistent Pneumocystis prophylaxis and risk-adapted surveillance during periods of profound immunosuppression. Tailored strategies that account for the dynamic immune status of patients with T/NKCL are needed to reduce IFI-associated mortality in this vulnerable population.

## Supplementary Material

Supplementary_263993423_R2.docx

## Data Availability

The datasets generated and analysed in the current study are available from the corresponding author upon reasonable request.

## References

[1] Denning DW. Global incidence and mortality of severe fungal disease. Lancet Infect Dis. 2024;24(7):e428–e38. doi: 10.1016/S1473-3099(23)00692-8. 38224705

[2] Koehler P, Hamprecht A, Bader O, et al. Epidemiology of invasive aspergillosis and azole resistance in patients with acute leukaemia: the SEPIA Study. Int J Antimicrob Agents. 2017;49(2):218–223. doi: 10.1016/j.ijantimicag.2016.10.019. 27989379

[3] Kontoyiannis DP, Marr KA, Park BJ, et al. Prospective surveillance for invasive fungal infections in hematopoietic stem cell transplant recipients, 2001–2006: overview of the Transplant-Associated Infection Surveillance Network (TRANSNET) Database. Clin Infect Dis. 2010;50(8):1091–1100. doi: 10.1086/651263. 20218877

[4] Alaggio R, Amador C, Anagnostopoulos I, et al. The 5th edition of the World Health Organization Classification of Haematolymphoid Tumours: lymphoid neoplasms. Leukemia. 2022;36(7):1720–1748. doi: 10.1038/s41375-022-01620-2. 35732829 PMC9214472

[5] Moskowitz AJ, Stuver RN, Horwitz SM. Current and upcoming treatment approaches to common subtypes of PTCL (PTCL, NOS; ALCL; and TFHs). Blood. 2024;144(18):1887–1897. doi: 10.1182/blood.2023021789. 38306597 PMC11830973

[6] Marchi E, Craig JW, Kalac M. Current and upcoming treatment approaches to uncommon subtypes of PTCL (EATL, MEITL, SPTCL, and HSTCL). Blood. 2024;144(18):1898–1909. doi: 10.1182/blood.2023021788. 38657272

[7] Ko T, Seah C, Gilbertson M, et al. A description of the type, frequency and severity of infections among sixteen patients treated for T-cell lymphoma. J Hematol. 2021;10(3):123–129. doi: 10.14740/jh838. 34267849 PMC8256920

[8] Charlson ME, Pompei P, Ales KL, et al. A new method of classifying prognostic comorbidity in longitudinal studies: development and validation. J Chronic Dis. 1987;40(5):373–383. doi: 10.1016/0021-9681(87)90171-8. 3558716

[9] Cheson BD, Fisher RI, Barrington SF, et al. Recommendations for initial evaluation, staging, and response assessment of Hodgkin and non-Hodgkin lymphoma: the Lugano classification. J Clin Oncol. 2014;32(27):3059–3068. doi: 10.1200/JCO.2013.54.8800. 25113753 PMC4979083

[10] International Non-Hodgkin’s Lymphoma Prognostic Factors P . A predictive model for aggressive non-Hodgkin’s lymphoma. N Engl J Med. 1993;329(14):987–994. 8141877 10.1056/NEJM199309303291402

[11] Ko B-S, Chen W-T, Kung H-C, et al. 2016 guideline strategies for the use of antifungal agents in patients with hematological malignancies or hematopoietic stem cell transplantation recipients in Taiwan. J Microbiol Immunol Infect. 2018;51(3):287–301. doi: 10.1016/j.jmii.2017.07.005. 28781151

[12] Donnelly JP, Chen SC, Kauffman CA, et al. Revision and Update of the Consensus Definitions of Invasive Fungal Disease From the European Organization for Research and Treatment of Cancer and the Mycoses Study Group Education and Research Consortium. Clin Infect Dis. 2020;71(6):1367–1376. doi: 10.1093/cid/ciz1008. 31802125 PMC7486838

[13] Pagano L, Akova M, Dimopoulos G, et al. Risk assessment and prognostic factors for mould-related diseases in immunocompromised patients. J Antimicrob Chemother. 2011;66 Suppl 1:i5–14. doi: 10.1093/jac/dkq437. 21177404

[14] Tisi MC, Hohaus S, Cuccaro A, et al. Invasive fungal infections in chronic lymphoproliferative disorders: a monocentric retrospective study. Haematologica. 2017;102(3):e108–e11. doi: 10.3324/haematol.2016.151837. 27856512 PMC5394967

[15] Axelrod PI, Lorber B, Vonderheid EC. Infections complicating mycosis fungoides and Sezary syndrome. JAMA. 1992;267(10):1354–1358. doi: 10.1001/jama.1992.03480100060031. 1740857

[16] Rayens E, Rayens MK, Norris KA. Demographic and socioeconomic factors associated with fungal infection risk, United States, 2019. Emerg Infect Dis. 2022;28(10):1955–1969. doi: 10.3201/eid2810.220391. 36149028 PMC9514344

[17] Egger M, Hoenigl M, Thompson GR, 3rd, et al. Let’s talk about sex characteristics: as a risk factor for invasive fungal diseases. Mycoses. 2022;65(6):599–612. doi: 10.1111/myc.13449. 35484713

[18] Takaoka K, Nannya Y, Shinohara A, et al. A novel scoring system to predict the incidence of invasive fungal disease in salvage chemotherapies for malignant lymphoma. Ann Hematol. 2014;93(10):1637–1644. doi: 10.1007/s00277-014-2093-1. 24908330

[19] Pappas PG, Lionakis MS, Arendrup MC, et al. Invasive candidiasis. Nat Rev Dis Primers. 2018;4(1):18026. doi: 10.1038/nrdp.2018.26. 29749387

[20] Cordonnier C, Cesaro S, Maschmeyer G, et al. Pneumocystis jirovecii pneumonia: still a concern in patients with haematological malignancies and stem cell transplant recipients. J Antimicrob Chemother. 2016;71(9):2379–2385. doi: 10.1093/jac/dkw155. 27550990

[21] Damaj G, Bazarbachi A, Berning P, et al. Allogeneic haematopoietic cell transplantation in peripheral T-cell lymphoma: recommendations from the EBMT Practice Harmonisation and Guidelines Committee. Lancet Haematol. 2025;12(7):e542-e54–e554. doi: 10.1016/S2352-3026(25)00073-0. 40610175

[22] Marchesini G, Nadali G, Facchinelli D, et al. Infections in patients with lymphoproliferative diseases treated with brentuximab vedotin: SEIFEM multicentric retrospective study. Leuk Lymphoma. 2020;61(12):3002–3005. doi: 10.1080/10428194.2020.1786562. 32611212

[23] Lionakis MS, Kontoyiannis DP. Glucocorticoids and invasive fungal infections. Lancet. 2003;362(9398):1828–1838. doi: 10.1016/S0140-6736(03)14904-5. 14654323

[24] Young J-AH, Andes DR, Ardura MI, et al. Modeling invasive Aspergillosis risk for the application of prophylaxis strategies. Open Forum Infect Dis. 2024;11(3):ofae082. doi: 10.1093/ofid/ofae082. 38481428 PMC10932941

[25] Kwong YL, Zhang H, Wang X, et al. Epidemiology of mature T-cell and NK-cell neoplasms: east and west. Lancet Reg Health West Pac. 2025;62:101646. doi: 10.1016/j.lanwpc.2025.101646. 41132163 PMC12541146

