# Supplementary material for: Epidemiology and outcomes of invasive fungal infections in patients with mature T-cell and NK-cell lymphomas
Source: Ann Med. 2026 Aug 26;58(1):2720365. doi: 10.1080/07853890.2026.2720365 (PMC13520847; doi:10.1080/07853890.2026.2720365)
Supplement: Supplementary file 1 [file IANN_A_2720365_SM8852.docx]

**Supplementary Appendix to** Epidemiology and Outcomes of Invasive Fungal Infections in Patients with Mature T-cell and NK-cell Lymphomas

***Author names and affiliations***

Ming-Tao Tsai^1^, Pao-Yu Chen^2*^, Tai-Chung Huang^3^, Jann-Tay Wang^2,4^, Wen-Chien Chou^3,5^, Yee-Chun Chen^2,4,5^

^1^ Department of Medicine, National Taiwan University Cancer Center, Taipei, Taiwan.

^2^ Division of Infectious Diseases, Department of Internal Medicine, National Taiwan University Hospital, Taipei, Taiwan.

^3^ Division of Hematology, Department of Internal Medicine, National Taiwan University Hospital, Taipei, Taiwan.

^4^ National Institute of Infectious Diseases and Vaccinology, National Health Research Institutes, Miaoli, Taiwan.

^5^ School of Medicine, National Taiwan University College of Medicine, Taipei, Taiwan.

**Supplementary Figure 1. Flowchart of the study population.**

**Supplementary Figure 2. Sankey plot of patient pathways stratified by treatment course, invasive fungal infection, and outcome.** Sankey diagram illustrating the clinical course of patients after lymphoma diagnosis. Flows depict progression from frontline lymphoma-directed therapy, with or without achievement of remission, to receipt or non-receipt of haematopoietic stem cell transplant, and subsequent development or absence of IFI during follow-up, culminating in survival or death. Node heights are proportional to the number of patients in each category.

**Supplementary Table 1. Distribution of mature T-cell and NK-cell lymphoma histologic subtypes and incidence of invasive fungal infection.**

| **Variable ^a^** | **Overall, N = 203** | **Any IFI, N = 43** | **Invasive yeast infection,  (22 episodes)** | **Invasive mould infection,  (15 episodes)** | ***Pneumocystis* *jirovecii* pneumonia,  (15 episodes)** |
| --- | --- | --- | --- | --- | --- |
| **Anaplastic large cell lymphoma** | 32 (16) | 3 (7) | 0 | 1 (7) | 2 (13) |
| **EBV-positive NK/T-cell lymphomas** | 35 (17) | 12 (28) | 7 (32) | 5 (33) | 4 (27) |
| **Hepatosplenic T-cell lymphoma** | 2 (1) | 2 (5) | 2 (9) | 0 | 1 (7) |
| **Intestinal T-cell and NK-cell lymphoid proliferations and lymphomas** | 7 (3) | 3 (7) | 3 (14) | 0 | 0 |
| **Mature T-cell and NK-cell leukaemias** | 3 (2) | 2 (5) | 1 (5) | 1 (7) | 1 (7) |
| **Nodal T-follicular helper cell lymphoma** | 41 (20) | 9 (21) | 3 (14) | 4 (27) | 4 (27) |
| **Other** *^b^* | 3 (2) | 1 (2) | 1 (5) | 0 | 0 |
| **Other peripheral T-cell lymphomas (PTCL, not otherwise specified)** | 29 (14) | 8 (19) | 4 (18) | 2 (13) | 3 (20) |
| **Primary cutaneous T-cell lymphomas** | 51 (25) | 3 (7) | 1 (5) | 2 (13) | 0 |

*^a^* n (%)

*^b^* Post-transplant lymphoproliferative disorder (n=2), and unknown subtype diagnosed from bone marrow biopsy (n=1).

**Abbreviations**: HSCT, haematopoietic stem cell transplant; IFI, invasive fungal infection.

**Supplementary Table 2. Sensitivity analyses for factors associated with invasive fungal infections: model A, those developing early IFI within 1 year after diagnosis of mature T-cell and NK-cell lymphomas; model B, those developing IFI before haematopoietic stem cell transplant.**

|  | **Model A: Early IFI** |  | **Model B: IFI before HSCT** |  |
| --- | --- | --- | --- | --- |
|  | **aOR (95% CI)** | ***P ^a^*** | **aOR (95% CI)** | ***P ^a^*** |
| **Male gender** | **5.83 (1.77 – 27.47)** | **0.007** | — | — |
| **IPI ≥2** | **4.75 (1.29 – 24.84)** | **0.033** | **6.20 (1.88 – 28.94)** | **0.007** |
| **Frontline lymphoma-directed therapy** | 2.33 (0.74 **–** 7.18) | 0.11 | 2.90 (0.71 **–** 19.76) | 0.19 |
| **Haemophagocytic lymphohistiocytosis** | 5.80 (1.00 – 110.35) | 0.14 | 2.74 (0.96 – 7.57) | 0.054 |
| **Gastrointestinal bleeding** | **19.02 (3.63 – 134.28)** | **<0.001** | **7.38 (1.86 – 31.69)** | **0.005** |

*^a^* Stepwise logistic regression; reference: those without invasive fungal infections.

**Abbreviations**: HSCT: haematopoietic stem cell transplant; IFI: invasive fungal infection; IPI, International Prognostic Index.

**Supplementary Table 3. Multivariable analyses for factors associated with each type of invasive fungal infections.**

|  | **Invasive yeast infection, aOR (95% CI)** | ***P ^a^*** | **Invasive mould infection, aOR (95% CI)** | ***P ^a^*** | ***Pneumocystis jirovecii* pneumonia, aOR (95% CI)** | ***P ^a^*** |
| --- | --- | --- | --- | --- | --- | --- |
| **Male gender** | — | — | 4.72 (1.05 – 31.46) | 0.066 | **13.24 (2.20 – 262.66)** | **0.020** |
| **Stage ≥2** | — | — | — | — | 8.89 (1.40 – 177.57) | 0.052 |
| **Haemophagocytic lymphohistiocytosis** | 2.53 (0.72 – 8.32) | 0.13 | — | — | — | — |
| **Disease remission after frontline lymphoma-directed therapy** | 0.46 (0.15 – 1.34) | 0.16 | 0.21 (0.03 – 0.91) | 0.055 | — | — |
| **Steroid** | — | — | 5.97 (0.69 – 171.16) | 0.17 | 5.25 (0.69 **–** 112.11) | 0.16 |
| **Gastrointestinal bleeding** | **7.27 (1.53 – 36.70)** | **0.012** | **11.50 (1.18 – 103.26)** | **0.025** | — | — |
| **Neutropenia** | **3.22 (1.11 – 10.89)** | **0.041** | — | — | — | — |
| **Trimethoprim–sulfamethoxazole prophylaxis** | — | — | — | — | **0.12 (0.02 – 0.49)** | **0.007** |
| **HSCT** | — | — | 3.09 (0.71 – 19.99) | 0.093 | **5.34 (1.18 – 26.64)** | **0.031** |
| **Acute graft-versus-host disease** | — | — | **16.64 (2.14 – 191.71)** | **0.012** | — | — |

*^a^* Stepwise logistic regression; reference: those without invasive fungal infections.

**Abbreviations**: HSCT: haematopoietic stem cell transplant; IFI: invasive fungal infection.

**Supplementary Table 4. Presentation of invasive mould infection.**

| **Variable** | **Invasive mould infection,  (15 episodes)** |
| --- | --- |
| **Chest CT (14 episodes of available imaging)** |  |
| Consolidation | 9 (64) |
| Nodules | 7 (50) |
| Cavitation | 1 (7) |
| Bilateral involvement | 9 (64) |
| Pleural effusion | 7 (50) |
| **Sinus CT (8 episodes of available imaging)** |  |
| Mucosal thickening | 6 (75) |
| Maxillary involvement | 4 (50) |
| Sphenoid involvement | 2 (25) |

**Supplementary Table 5. Frontline lymphoma-directed therapy categories for mature T-cell and NK-cell lymphoma**

| **Frontline lymphoma-directed therapy categories** | **N (%)** |
| --- | --- |
| Anthracycline-based CHOP-like regimens ^a^ | 99 (48.8) |
| Asparaginase-containing regimens ^b^ | 15 (7.4) |
| Concurrent chemoradiotherapy-based approaches ^c^ | 13 (6.4) |
| Bendamustine-containing regimens | 6 (3.0) |
| Other regimens ^d^ | 5 (2.5) |
| Platinum-based regimens ^e^ | 3 (1.5) |

^a^ Anthracycline-based CHOP-like regimens included CHOP (cyclophosphamide, hydroxydaunorubicin, vincristine sulfate, and prednisone), CHOEP (cyclophosphamide, hydroxydaunorubicin, vincristine sulfate, etoposide, and prednisone) or L-CHOP (L-asparaginase, cyclophosphamide, hydroxydaunorubicin, vincristine sulfate, etoposide, and prednisone).

^b^ Asparaginase-containing regimens included AspaMetDex (asparaginase, methotrexate, and dexamethasone), DDGP (pegaspargase, gemcitabine, cisplatin and dexamethasone), GELOX (gemcitabine, L-asparaginase, and oxaliplatin), GEMOX (gemcitabine and oxaliplatin) and SMILE (steroid, methotrexate, ifosfamide, L-asparaginase, and etoposide).

^c^ Concurrent chemoradiotherapy-based approaches included RT-2/3DeVIC (dexamethasone, etoposide, ifosfamide, and carboplatin) and VIPD (etoposide, ifosfamide, cisplatin, and dexamethasone).

^d^ Other regimens included gemcitabine-based chemotherapies, etoposide monotherapy and HDMTX (high-dose methotrexate).

^e^ Platinum-based regimens included ICE (ifosfamide, carboplatin, and etoposide), DHAP (dexamethasone, cytarabine, and cisplatin), and ESHAP (etoposide, methylprednisolone, cytarabine, and cisplatin).

**Supplementary Table 6. Treatment and outcomes of invasive fungal infection.**

| **Variable** | **Invasive yeast infection,  (22 episodes)** | **Invasive mould infection,  (15 episodes)** | ***Pneumocystis* *jirovecii* pneumonia,  (15 episodes)** |
| --- | --- | --- | --- |
| **Primary agents ^a^** |  |  |  |
| Fluconazole | 1 (5) *^b^* | NA | NA |
| Voriconazole | 0 | 9 (60) | NA |
| Isavuconazole | 0 | 4 (27) | NA |
| Echinocandin | 13 (59) | 0 | NA |
| Liposomal amphotericin B & voriconazole | 0 | 1 (7) | NA |
| Liposomal amphotericin B & flucytosine | 2 (9) *^c^* | 0 | NA |
| Trimethoprim–sulfamethoxazole | NA | NA | 12 (80) |
| **Salvage agents** | NA | 5 (33) | NA |
| Isavuconazole | NA | 4 (27) | NA |
| Liposomal amphotericin B | NA | 1 (7) | NA |
| **No treatment *^d^*** | 6 (27) | 1 (7) | 3 (20) |
| **Outcomes** |  |  |  |
| 14-day mortality | 11 (50) | 4 (27) | 4 (27) |
| 28-day mortality | 13 (59) | 7 (47) | 7 (47) |
| 42-day mortality | 15 (68) | 9 (60) | 8 (53) |
| 84-day mortality | 17 (77) | 12 (80) | 9 (60) |

*^a^* n (%)

*^b^* For *Trichosporon* fungemia.

*^c^* For Cryptococcosis.

*^d^* These patients passed away prior to the availability of culture reports and/or mycology data, so they did not receive targeted antifungal therapy.
